# Reduced Central Memory CD4+ T Cells and Increased T-Cell Activation Characterise Treatment-Naive Patients Newly Diagnosed at Late Stage of HIV Infection

**DOI:** 10.1155/2012/314849

**Published:** 2011-10-27

**Authors:** Francesca Bai, Camilla Tincati, Esther Merlini, Carlotta Pacioni, Elisabetta Sinigaglia, Giovanni Carpani, Antonella d'Arminio Monforte, Giulia Marchetti

**Affiliations:** ^1^Department of Medicine, Surgery and Dentistry, Clinic of Infectious Diseases and Tropical Medicine, S. Paolo Hospital, University of Milan, via A. di Rudinì, 8-20142 Milan, Italy; ^2^Blood Transfusion Centre, San Paolo Hospital, Milan, Italy

## Abstract

*Objectives*. We investigated immune phenotypes of HIV+ patients who present late, considering late presenters (LPs, CD4+ < 350/**μ**L and/or AIDS), advanced HIV disease (AHD, CD4+ < 200/**μ**L and/or AIDS), and AIDS presenters (AIDS-defining condition at presentation, independently from CD4+). *Methods*. Patients newly diagnosed with HIV at our clinic between 2007–2011 were enrolled. Mann-Whitney/Chi-squared tests and logistic regression were used for statistics. *Results*. 275 patients were newly diagnosed with HIV between January/2007–March/2011. 130 (47%) were LPs, 79 (29%) showed AHD, and 49 (18%) were AIDS presenters. LP, AHD, and AIDS presenters were older and more frequently heterosexuals. Higher CD8+%, lower CD127+CD4+%, higher CD95+CD8+%, CD38+CD8+%, and CD45R0+CD38+CD8+% characterized LP/AHD/AIDS presentation. In multivariate analysis, older age, heterosexuality, higher CD8+%, and lower CD127+CD4+% were confirmed associated with LP/AHD. Lower CD4+ and higher CD38+CD8+% resulted independently associated with AIDS presentation. *Conclusions*. CD127 downregulation and immune activation characterize HIV+ patients presenting late and would be studied as additional markers of late presentation.

## 1. Introduction

Despite reduced morbidity and mortality achieved by highly active antiretroviral therapy (HAART) and extensive work encouraging earlier HIV testing, late presentation of HIV remains a relevant clinical problem. Up to 15%–38% of HIV positive patients present late for testing with a low CD4+ T-cell count, high viral load, and severe alterations of their immune system [1]. Focusing on the Italian scenario, 39% of new HIV diagnosis occur late [2]. Compared to patients diagnosed with HIV early in the course of infection, late presenters are at higher probability of clinical progression and treatment-related adverse events and introduce HAART later than recommended by current antiretroviral therapy guidelines [3, 4]. Furthermore, late presentation deeply impacts on healthcare system and community in terms of resource use and higher risk of HIV transmission to sexual partners [5]. Recently, two different classifications of late presentation have been established by the European AIDS Clinical Society [6]: all patients who present with a CD4+ T-cell count less than 350 cells/*μ*L and/or an AIDS-defining condition at, or within a month after, HIV diagnosis are identified as “late presenters.” This definition allows for the identification of patients that should be considered for treatment in accordance with current guidelines. Secondly, focusing on the established risk cutoff for developing opportunistic infections, all patients who present with a CD4+ T-cell count less than 200 cells/*μ*L and/or an AIDS event should be considered to have “advanced HIV disease” [7, 8].

Previous studies identified the demographic parameters associated with a high risk of late presentation. Older age, heterosexual transmission of infection, and foreign birth (non-White and in particular Black African ethnicity) may influence the risk of late diagnosis [1–13]. On the contrary, a clear association between gender and late HIV testing has not been recognized [9–14]. 

As expected, low CD4+ T-cell count at presentation associates with increased risk of AIDS defining illnesses. The most commonly diagnosed AIDS conditions are *Pneumocystis jiroveci* pneumonia and tuberculosis, followed by Cytomegalovirus infections, toxoplasmosis, and wasting syndrome. Patients with AIDS at presentation represent a real medical challenge because of their advanced clinical status and higher risk of clinical progression and mortality. At least two clinical challenges relate to these patients and include the timing of HAART introduction based on the concomitant opportunistic infections and the elevated risk of failing a satisfactory CD4+ recovery, even after long-term virological suppression by HAART [15, 16].

A broad immunological characterization of patients with a late diagnosis of HIV infection and/or an AIDS disease at presentation is still lacking. Detailing the alterations of immune system homeostasis associated to late presentation would allow for the identification of an early immunological marker to complement CD4+ T-cell count in the clinical outcome of late presenter patients; this could be crucial in order to tailor antiretroviral therapy to individual circumstances of late presenters.

In this context, the aim of this study is to analyze the peripheral T lymphocyte phenotypes in patients who were newly diagnosed with HIV and to determine demographic and immunological factors associated with late HIV positive testing, defined in accordance with the two most recent classifications (late presentation and advanced HIV disease).

## 2. Materials and Methods

### 2.1. Study Design and Population

We identified all patients aged >18 years who were newly diagnosed with HIV at the Clinic of Infectious Diseases of “S. Paolo” Hospital in Milan, Italy, between January 2007 and March 2011. Analyses were restricted to subjects with a new HIV antibody-positive test and at least one CD4+ T-cell count within 30 days of HIV diagnosis. Persons with a previous positive HIV test were excluded from the analysis.

Information on demographic parameters (sex, date of birth, country of birth) and HIV-related data (HIV exposure category, calendar period of HIV diagnosis, AIDS event, CD4+ T-cell count, HIV-RNA, and HCV coinfection) at presentation were retrospectively collected. 

We defined “migrants” patients who were born outside the European Community (including people from Eastern Europe, Africa, Asia, and Latin America).

### 2.2. Definitions of Late Presentation

We used two different definitions of late presentation, in line with the recent indications by UK Collaborative HIV Cohort (CHIC) study and HIV in Europe group [6, 7]. Definitions were not mutually exclusive.

According to the first classification, all patients whose CD4+ T-cell count at the time of diagnosis was <350 cells/*μ*L were defined “late presenters” (LPs). Secondly, all patients who present with a CD4+ T lymphocyte count <200 cells/*μ*L were considered to have “advanced HIV disease” (AHD). In all definitions, patients presenting with an AIDS defining condition were considered as LP and AHD subjects regardless CD4+ T-cell count. Patients with an AIDS diagnosis at presentation (defined according to the 1993 Centers for Disease Control and Prevention criteria) [17] from 30 days prior to 30 days after their first positive HIV test were considered as AIDS presenters.

### 2.3. T-Cells Immune Phenotypes

Peripheral T lymphocyte immune phenotypes were recorded for all patients. Lymphocyte surface phenotypes were evaluated by flow cytometry on fresh peripheral blood (Coulter ESP; Beckman Coulter, Hialeah, Fla) using the following fluorochrome-labelled antibodies: CD4-Pcy7, CD8-Pcy5, CD38-FITC, CD45-ECD, CD45R0-PE, CD95-FITC, and CD127-PE. Percentages of each T-cell subpopulations were calculated on whole lymphocyte population. We evaluated activation (expression of CD45R0 and CD38 on CD8+), sensitivity to Fas-mediated cells death (expression of CD95 on CD4+ and CD8+), and IL-7 receptor (CD127) expression on CD8+ and CD4+ T cells. The following combinations were used: CD8/CD38, CD8/CD38/CD45R0, CD8/CD4/CD95, and CD8/CD4/CD127.

### 2.4. Statistical Analysis

Continuous variables were expressed as median and interquartile range (IQR), categorical variables as absolute numbers and percentages. Baseline differences between LPs, and nonlate presenters (N-LPs, patients with CD4+ T-cell count ≥350 cells/*μ*L and without an AIDS diagnosis at presentation), between AHD subjects and patients who did not display advanced HIV disease (N-AHD, CD4+ T-cell count ≥200 cells/*μ*L and without an AIDS event at presentation), and finally between AIDS presenters and patients asymptomatic for AIDS diseases at presentation (N-AIDS presenters) were assessed using the Mann-Whitney nonparametric *U*-test and the Chi-squared test for continuous and categorical variables, respectively.

In addition, we performed further analyses using N-LP as unique comparison group.

Multivariate logistic regression models were used to identify baseline peripheral immune phenotypes that were independently associated with LP, AHD, or AIDS presentation. T lymphocyte phenotypes with a *P* value ≤0.05 in the univariate analyses entered the final multivariate models. Each final model was adjusted for potential confounders: the models were adjusted for the demographic parameters that resulted significantly associated with the outcome in the univariate analysis. 

Analyses were performed using SPSS software (version 18.01). A *P* value ≤0.05 was considered to denote statistical significance.

## 3. Results

### 3.1. Baseline Characteristics of the Study Population

During the study period (January 2007–March 2011), 275 patients were diagnosed for the first time with HIV infection at our clinic. Characteristics of the 275 subjects at presentation are summarized in Table 1.

Overall, the majority of patients were male (225/275, 82%) with a median age of 37 (IQR 31–46) years and acquired HIV infection mostly through sexual contacts (homosexual transmission 152/275, 55%—heterosexual transmission 111/275, 40%) (Table 1(a)).

A total of 16 (6%) patients displayed HCV coinfection. Most subjects were of white ethnicity, while 73 (26%) HIV-positive patients were born outside the European community. 

The proportion of new HIV diagnosis remained stable over time (31% in 2007, 22% in 2008-2009-2010).

### 3.2. Immunovirological Parameters of the Study Population

Median CD4+ count at first presentation for HIV care was 396 cells/*μ*L (IQR 234–555), and median viral load was 4.37 log_10_ cp/mL (IQR 2.78–5.16). The other percentages (median, IQR) of peripheral T lymphocytes immune phenotypes were reported in Table 1(b).

### 3.3. Characteristics of Late Presenters

130 (47%) patients showed CD4+ T-cell counts <350 cells/*μ*L and/or an AIDS defining event at presentation and were classified as late presenters (LPs) (Table 2). Compared to N-LPs, LPs were older (median age: LP 41, IQR 34–50—N-LP 36, IQR 30–43 years; *P* = 0.0001), contracted HIV infection more frequently through heterosexual contacts (heterosexual transmission: LP 64, 49%—N-LP 47, 32%; homosexual transmission: LP 59, 45%—N-LP 93, 64%; intravenous drug users: LP 6, 5%—N-LP 4, 3%; *P* = 0.020), and resulted more commonly migrants (LP 43, 33%—N-LP 30, 21%; *P* = 0.020). No differences were observed between the two groups of patients regarding gender, coinfections, and calendar year of presentation (Table 2(a)).

Interestingly, analyzing the immune phenotypes of peripheral T lymphocytes, we found that LP were characterized by a significantly different immunological pattern in comparison to N-LP. In particular, LP displayed higher CD8+ T-cells percentages (LP 57, IQR 51–63—N-LP 45, IQR 38–54; *P* = 0.0001), lower IL-7 receptor (CD127) expression on CD4+ T cells (median CD127+CD4+%: LP 7, IQR 4–12—N-LP 19, IQR 12–24; *P* = 0.0001), and higher expression of CD95 receptor (median CD95+CD8+%: LP 3, IQR 2–5—N-LP 2, IQR 1–3; *P* = 0.0001), activated CD38+ (median CD38+CD8+%: LP 9, IQR 3–18—N-LP 5, IQR 2–9; *P* = 0.0001) and terminal-differentiated CD45R0+CD38+ (median CD45R0+CD38+CD8+%: LP 15, IQR 10–25—N-LP 9, IQR 5–16; *P* = 0.0001) on CD8+ T cells. The other immune phenotypes did not result significantly different between LP and N-LP (Table 2(b)).

### 3.4. Characteristics of Patients Presenting Advanced HIV Disease

Using the second criteria to identify patients with a new diagnosis of HIV infection in a late stage of disease, 79 (29%) subjects presented advanced HIV disease (AHD) (Table 3). Similarly to the first definition, AHD patients resulted older (median age: AHD 42, IQR 34–52—N-AHD 36, IQR 31–44; *P* = 0.0001) and more frequently heterosexual (heterosexual transmission: AHD 41, 52%—N-AHD 70, 36%; homosexual transmission: AHD 34, 43%—N-AHD 118, 60%; intravenous drug users: AHD 4, 5%—N-AHD 6, 3%; *P* = 0.044). The proportion of HIV/HCV coinfection and migrants were comparable between the two groups of patients; in parallel, from 2007 to the first months of 2011, we did not register a substantial decrease of late diagnosis (Table 3(a)). 

Diverse immune phenotype patterns were detected in AHD and N-AHD patients: AHD presented higher CD8+ (AHD: 59, IQR 51–66—N-AHD 48, IQR 41–57; *P* = 0.0001), lower CD127+CD4+ (median CD127+CD4+%: AHD 6, IQR 3–11—N-AHD 15, IQR 10–22; *P* = 0.0001), higher CD95+CD8+ (median CD95+CD8+%: AHD 3, IQR 2–6—N-AHD 2, IQR 1–3; *P* = 0.0001), CD38+CD8+ (median CD38+CD8+%: AHD 11, IQR 3–25—N-AHD 5, IQR 2–10; *P* = 0.0001), and CD45R0+CD38+CD8+ (median CD45R0+CD38+CD8+%: AHD 14, IQR 10–25—N-AHD 11, IQR 7–19; *P* = 0.008) percentages in comparison to N-AHD (Table 3(b)).

### 3.5. AIDS Presenters

An AIDS defining condition was reported for 49/275 (18%) subjects with a new HIV diagnosis during the study period. The most common diseases were Kaposi's Sarcoma (12/49, 24%), *Pneumocystis jiroveci* pneumonia (8/49, 16%), and tuberculosis (7/49, 14%). Other AIDS-related illnesses were CMV infection (sepsis, chorioretinitis, and colitis, 4/49, 8%), neurological diseases (HIV-associated dementia, “HAD,” progressive multifocal leukoencephalitis, “PML,” and neurotoxoplasmosis, 7/49, 14%), lymphomas (4/49, 8%), esophageal candidiasis (3/49, 7%), Wasting syndrome (3/49, 7%), and cervical cancer (1/49, 2%).

AIDS presenters were more frequently older (median age: AIDS presenters: 42, IQR 35–52—N-AIDS presenters 37, IQR 31–44; *P* = 0.0001), male (AIDS presenters: male 45, 92%—female 4, 8%; N-AIDS presenters: male 180, 80%—female 46, 20%; *P* = 0.045), and heterosexuals (heterosexual transmission: AIDS presenters 29, 59%—N-AIDS presenters 82, 36%; homosexual transmission: AIDS presenters 17, 35%—N-AIDS presenters 135, 60%; intravenous drug users: AIDS presenters 3, 6%—N-AIDS presenters 7, 3%; *P* = 0.044), compared to N-AIDS presenters (226 subjects).

Comparing immune phenotypes between AIDS presenters and N-AIDS presenters, we observed that AIDS presenters were associated with lower CD4+ T-cell count (median CD4+ cells/*μ*L: AIDS presenters 132, IQR 60–286—N-AIDS presenters 438, IQR 286–583; *P* = 0.0001), and higher CD8+ T cells (median CD8+%: AIDS presenters 59, IQR 50–66—N-AIDS presenters 49, IQR 42–59; *P* = 0.0001). AIDS presenters featured lower CD127+CD4+% (median CD127+CD4+%: AIDS presenters 6, IQR 4–11—N-AIDS presenters 14, IQR 8–21; *P* = 0.0001), higher CD95+CD8+% (median CD95+CD8+%: AIDS presenters 3, IQR 2–6—N-AIDS presenters 1, IQR 2–4; *P* = 0.003), CD38+CD8+% (median CD38+CD8+%: AIDS presenters 12, IQR 5–23—N-AIDS presenters 6, IQR 2–11; *P* = 0.0001), and CD45R0+CD38+CD8+% (median CD45R0+CD38+CD8+%: AIDS presenters 16, IQR 10–25—N-AIDS presenters 11, IQR 7–19; *P* = 0.02).

### 3.6. Factors Independently Associated with Late Presentation

Given that LPs were distinguished by a peculiar peripheral immune phenotype, we investigated the association between T lymphocyte patterns and late presentation, controlling for potentially confounding factors. We performed a logistic regression model (Table 4) including the peripheral immune phenotypes that resulted significantly associated with LP in the univariate analysis (CD8+%, CD4+CD127+%, CD8+CD95+%, CD8+CD38+%, and CD8+CD38+CD45R0+%) (Table 2(b)), mutually adjusting for demographic factors (age, exposure category for HIV, ethnicity). Higher CD8+ T-cell percentages (AOR 1.051 for each unit more, 95%CI 1.022–1.080, *P* = 0.0001) and lower CD127+ expression on CD4+ cells (AOR 0.887 for each unite more, 95%CI 0.857–0.919, *P* = 0.0001) resulted significantly associated with late presentation, independently of demographic factors.

As expected, also demographic factors were significantly and independently associated with late presentation; in fact, older age (AOR 1.052 for each year more, 95%CI 1.020–1.086, *P* = 0.001), heterosexual acquisition of infection (AOR 2.435 versus other risk groups, 95%CI 1.251–4.741, *P* = 0.009), and non-White race (AOR 2.543 versus white ethnicity, 95%CI 1.178–5.486, *P* = 0.017) were associated with higher risk of delayed HIV testing.

### 3.7. Factors Independently Associated with Advanced HIV Disease

To identify possible factors associated with AHD, significant T-cells immune phenotypes in the univariate analysis (CD8+%, CD4+CD127+%, CD8+CD95+%, CD8+CD38+%, and CD8+CD38+CD45R0+%) (Table 3(b)) were entered in a logistic regression model, mutually adjusted for age and risk group (Table 5).

The immune phenotypes independently associated with AHD, after controlling for age and risk groups, were higher CD8+% (AOR 1.051 for each unit more, 95%CI 1.022–1.081, *P* = 0.001), lower CD127+CD4+% (AOR 0.909 for each unit more, 95%CI 0.878–0.942, *P* = 0.0001), and, in this case, also higher-activated CD38+CD8+% (AOR 1.065 for each unit more, 95%CI 1.020–1.112, *P* = 0.004) and lower terminal-differentiated CD45R0+CD38+CD8+% (AOR 0.955 for each unit more, 95%CI 0.916–0.996, *P* = 0.032). AHD patients were confirmed again significantly characterized by older age than N-AHD subjects (AOR 1.040 for each year more, 95%CI 1.010–1.071, *P* = 0.008).

### 3.8. Factors Independently Associated with AIDS Presentation

To explore eventual immune phenotypes specifically characterizing patients with an AIDS-defining pathology at new diagnosis of HIV, we also conducted a multivariate logistic regression analysis considering AIDS presentation (Table 6). Also, in this case, the parameters with a *P* value ≤0.05 in the univariate analysis entered the logistic regression model: the covariates were CD4+%, CD8+%, CD4+CD127+%, CD8+CD95+%, CD8+CD38+%, and CD8+CD38+CD45R0+%, and the model was adjusted for age, gender, and risk group.

The immunological patterns associated with AIDS presentation independently of demographic factors were of course lower CD4+% T cells (AOR 0.996 for each unit more, 95%CI 0.993–0.998, *P* = 0.008) but also higher CD38+CD8+% (AOR 1.049 for each unit more, 95%CI 1.004–1.096, *P* = 0.033). In addition, AIDS presenters were confirmed significantly associated with heterosexual risk group (AOR 4.555 versus other risk categories, 95%CI 1.937–10.711, *P* = 0.001) and male gender (AOR 9.369 versus female, 95%CI 2.401–36.551, *P* = 0.001).

### 3.9. Comparison between AHD Subjects/AIDS Presenters and N-LP

We further compared the 79 AHD subjects and 49 AIDS presenters each with the 145 N-LP (comparison group). 

AHD patients and AIDS presenters resulted older (median age: N-LP 36, IQR 30–43 versus AHD 42, IQR 34–52, *P* = 0.0001; versus AIDS presenters 42, IQR 35–52, *P* = 0.0001) and more frequently heterosexually infected (heterosexuals: N-LP 47, 32% versus AHD 41, 52%, *P* = 0.016; versus AIDS presenters 29, 59%, *P* = 0.003) in comparison to N-LP (see Supplementary Material Table 1 available online at doi: 10.1155/2012/314849). 

As concern peripheral T lymphocyte immune phenotypes, compared to N-LP, AHD patients and AIDS presenters were characterized by higher CD8+% (NLP: 45, 38–45 versus AHD: 59, 51–66, *P* = 0.0001; versus AIDS presenters: 59, 50–66, *P* = 0.0001), lower CD127+CD4+% (NLP: 19, 12–24 versus AHD: 6, 3–11, *P* = 0.0001; versus AIDS presenters: 6, 4–11, *P* = 0.0001), higher CD95+CD8+% (NLP: 2, 1–3 versus AHD: 3, 2–6, *P* = 0.0001; versus AIDS presenters: 3, 2–6, *P* = 0.0001), CD38+CD8+% (NLP: 5, 2–9 versus AHD: 11, 3–25, *P* = 0.0001; versus AIDS presenters: 12, 5–23, *P* = 0.0001), and CD45R0+CD38+CD8+% (NLP: 9, 5–16 versus AHD: 14, 10–25, *P* = 0.0001; versus AIDS presenters: 16, 10–25, *P* = 0.0001) (Supplementary Table 1).

Finally, we performed two different logistic regression models to assess eventual independent markers of AHD and AIDS presentation, respectively, always using N-LP as the unique comparison group.

Interestingly, CD8+% (AOR 1.061 for each unit more, 95%CI 1.031–1.093, *P* = 0.0001), CD8+CD38+% (AOR 1.066 for each unit more, 95%CI 1.009–1.126, *P* = 0.022), CD8+CD38+CD45R0+% (AOR 0.955 for each unit more, 95%CI 0.993–0.997, *P* = 0.0001), and CD4+CD127+% (AOR 0.837 for each unit more, 95%CI 0.792–0.884, *P* = 0.0001) were significantly associated with AHD, also after controlling for age and risk groups for HIV infection (Supplementary Table 2).

Indeed, AHD subjects were confirmed older than N-LP (AOR 1.051 for each year more, 95%CI 1.012–0.976, *P* = 0.010) also in the multivariate analysis (Supplementary Table 2).

Similarly, we observed an independent association between AIDS presentation and CD8+CD38+% (AOR 1.078 for each unit more, 95%CI 1.015–1.146, *P* = 0.015), CD8+CD38+CD45R0+% (AOR 0.996 for each unit more, 95%CI 0.994–0.999, *P* = 0.004), and CD4+CD127+% (AOR 0.827 for each unit more, 95%CI 0.759–0.902, *P* = 0.0001). Also, this model was adjusted for age and exposure category for HIV transmission, and, as expected, for each year more of age (AOR 1.065 for each year more, 95%CI 1.019–1.113, *P* = 0.005) and for heterosexual transmission (AOR 3.176 versus other risk categories, 95%CI 1.182–8.531, *P* = 0.022), we reported a significant increase of risk of AIDS presentation.

## 4. Discussion

In our clinic, we observed 275 new HIV diagnosis in the period 2007–2011 with no significant differences over time. Still nowadays, the greatest percentages of persons diagnosed with HIV/AIDS are men, of minority ethnicity, and have as principal HIV transmission risk factor sexual contacts. Globally, in our experience, patients who present a new HIV-positive test display a median CD4+ T cells at presentation of 396 cells/*μ*L and, therefore, near the minimum CD4+ count threshold for initiation of HAART, as suggested by the most recent international guidelines [6, 18–20]. In fact, different studies have demonstrated that initiating therapy at CD4+ levels higher than 350 cells/*μ*L improves survival [21]. Furthermore, patients with a CD4+ count between 350 and 500 cells/*μ*L and a HIV-RNA >100.000 copies/mL should be considered for treatment [6]. For this reason, encouraging an earlier recourse to HIV testing and access to care becomes crucial.

Across Europe, many patients still present late in the course of infection [2]. A universal standard definition of late presentation has not still found, even if a common classification could facilitate cross-country comparisons, identification of risk factors for late testing in different settings and trends in the rate of late presentation over time. A recent work by UK Collaborative Cohort (UK CHIC) has proposed two definitions: “late presentation” (LP), presentation when CD4+ cell count is below the limit of antiretroviral therapy introduction (current CD4+ <  350 cells/*μ*L and/or a clinical AIDS event at HIV diagnosis); “advanced HIV disease” (AHD), presentation associated with a substantial increase in clinical progression/death risk (current CD4+ <  200 cells/*μ*L and/or AIDS event) [7].

In our study, we reported a prevalence of 47% of LP and 29% of AHD, in line with recent Italian surveys [22, 23]. Regarding demographic parameters associated with LP and AHD, we confirm that older age and foreign birth outside European community are risk factors for late HIV testing, as already widely demonstrated in previous studies [13]. Indeed, LP and AHD were more frequently heterosexuals, even if absolute numbers of homosexual people presenting late remain high because of the high prevalence of homosexuals among those HIV infected [22–25]. We observed few cases of LP/AHD among intravenous drug users (IDUs): this scenario is confirmed by other Italian studies that did not find an association between late presentation, and IDUs [11]. On the contrary, in other European works, the proportion of IDUs who were diagnosed for HIV infection late reached 38.7%, but this difference could reflect dissimilar screening policies among IDUs [26].

We did not observe differences in gender between LP/AHD and N-LP/N-AHD: in literature, there are some works that found an association between males and late HIV testing, while others described an increased risk of late presentation in women, particularly migrants [11, 14, 15].

We also recorded a high but stable proportion of LP/AHD in the study period (2007–2011); previous literature reports described the same temporal trends [27], but other studies conducted on larger population observed a decline in the numbers of patients presenting with advanced disease in 2000–2010 [28]. 

We then focused our attention on the immunological phenotype of T lymphocytes in peripheral blood associated with late presentation. As expected, we found that LP and AHD were characterized by higher percentages of CD8+ T cells, also in the multivariate analysis. It is known that during HIV infection, persistent viral replication determines immune perturbation with high levels of CD8+ T-cells activation/turnover [29]. Interestingly, LP/AHD also displayed significantly lower percentages of CD127 on the CD4+ pool. The expression of interleukin- (IL-) 7 receptor (CD127, IL-7R) and the activity of IL-7/CD127 system are central for the generation, survival, and differentiation of naïve and central memory T cells; in fact, perturbations in this system are linked to faster disease progression in acute and chronic HIV infection [30, 31]. Downregulation of IL-7R is also associated with T-cell activation and immune recovery following HAART [32]; we recently described that CD127 expression on CD4+ T cells was the only marker associated with a reduced risk of incomplete immune response under HAART [33]. Loss of IL-7R on T cells seems to determine an impairment of these cells to response to IL-7 with subsequent inability to differentiation or reversion to resting central memory phenotype and a generalized immune activation with increased susceptibility to apoptosis [29]. In fact, LP and AHD subjects were characterized also by higher CD8+ expression of the death receptor Fas (CD95+), activated CD38+CD8+ T cells, and terminally differentiated CD45R0+CD38+CD8+ T cells, compared to N-LP and N-AHD. It has been previously demonstrated how CD38+CD8+ phenotype has predictive value for progression to AIDS, even after adjustment for CD4+ levels, and it has been proposed as surrogate marker to clinical monitoring of HIV infection [34, 35]. Again, in our previous work [33], the proportion of CD38+CD8+ T cells resulted a feature of patients introducing late HAART together with higher levels of CD8+ cells with increased vulnerability to apoptosis (i.e., CD95+) and lower CD127+CD4+ T cells. Indeed, increased numbers of primed activated CD8+CD38+CD45R0+ T cells have been shown to predict the decline of CD4+ T cells in HIV-1-infected patients [36, 37] and can be considered a marker of immune exhaustion reflecting a history of elevated rounds of proliferation [38]. Although Fas expression on peripheral T cells are not indicative of an active apoptotic process, we believe that our data suggest an increased sensitivity of circulating CD8+ T lymphocytes to Fas-mediated cells death.

On the basis of previous literature findings, we could hypothesise that HIV-infected patients with a first HIV diagnosis late in the course of infection display a highly dysregulated immunity with expansion of activated/senescent T-cell phenotypes and contraction of central memory cells; in this context, loss of CD127 on T cells following immune activation determines a proapoptotic signal with reinforcement of CD4 declining and disease progression.

Furthermore, multivariate analysis confirmed reduction of CD127 on CD4+ T cells as the main marker of late presentation; given the positive effects of IL-7 on homeostatic survival and proliferation of T cells, lower CD127+CD4+ percentages could be used as additional marker, together with CD4+ counts, to identify HIV-positive patients in a particular advanced stage of disease and at higher risk of clinical progression and death.

Finally, we studied the demographic factors and immune phenotypes characterizing patients presenting with AIDS-defining conditions. Among LP/AHD, the proportion of AIDS presenters has increased over time and the immunological status at presentation is severely compromised with a CD4+ count frequently <50 cells/*μ*L [3]. Overall, also in our population, almost 20% (49/275, 18%) of patients were AIDS presenters. As expected, they were older and more frequently heterosexuals than patients without an AIDS event. In this case, patients with AIDS resulted also more commonly male, but globally our population presented a higher prevalence of male subjects.

From an immunological standpoint, these patients showed again a deeply compromised immune system: in addition to lower CD4+ and higher CD8+ levels, we observe a downregulation of CD127 on CD4+ T cells, higher expression of CD95+ on CD8+ T cells and higher activated CD38+CD8+, and terminal-differentiated CD45R0+CD38+CD8+ T cells, probably reflecting the same perturbations of immunological homeostasis registered in late presentation. In multivariate analysis, however, besides declining CD4+ cells, the main association with AIDS presentation was the presence of higher immune activation, consistent with the notion that in AIDS patients a profound immunodeficiency strongly correlates with generalized immune activation.

## 5. Conclusion

A broader definition of immunological features of late presenters could permit a better identification of patients subsets at high risk of clinical progression and reduced immunological response to HAART. Besides CD4+ levels, CD127+ expression on CD4+ T lymphocytes, and T-cell activation could be proposed as novel additional markers of late presentation and advanced HIV disease. Further studies on larger population are needed to confirm immunological patterns associated with late presentation.

## Supplementary Material

In the Supplementary table 1 we compared demographic parameters and peripheral T lymphocytes immune phenotypes between N-LP and AHD patients (a-c)/AIDS presenters (b-d).
The results of the multivariate logistic regression are represented in Supplementary Table 2; we assessed two logistic regression models to explore eventual factors independently assoociated with AHD (model a) and AIDS presentation (model b), using N-LP as the unique comparison group. Significant immune phenotypes in the univariate analysis (CD8*+*%, CD4*+*CD127*+*%, CD8*+*CD95*+*%, CD8*+*CD38*+*%, CD8*+*CD38*+*CD45R0*+*%) entered the multivariate regression models, adjusted for age and risk group for HIV infection.

## Figures and Tables

**Table:** (a)

Characteristics	Patients (*N* = 275)
Age, years*	37 (31–46)
Gender, male°	225 (82)
Risk group°	
Homosexual	152 (55)
Heterosexual	111 (40)
IDUs	10 (4)
Other	2 (1)
Migrants°	73 (26)
HCV coinfection°	16 (6)
Calendar year of presentation°	
2007	85 (31)
2008	61 (22)
2009	61 (22)
2010	61 (22)
January–March 2011	7 (3)
AIDS presenters°	49 (18)
HIV-RNA log_10_ cp/mL*	4.37 (2.78–5.16)
CD4+ T cells/*μ*L*	396 (234–555)

**Table:** (b)

T-cells immunophenotypes	Patients (*N* = 275)
CD4 T cells %*	22 (14–30)
CD8 T cells %*	51 (42–60)
CD4+CD127+ cells %*	12 (7–20)
CD8+CD127+ cells %*	11 (8–17)
CD4+CD95+ cells %*	2 (1–4)
CD8+CD95+ cells %*	2 (1–4)
CD8+CD38+ cells %*	6 (3–12)
CD8+CD38+CD45R0+ cells %*	12 (7–20)

Data are presented as *median, (interquartile range, IQR) and °absolute number, (%). IDUs: injection drug users; migrants: people who were born outside the European community (including people from Eastern Europe, Africa, Asia, and Latin America); HCV: hepatitis C virus.

**Table:** (a)

Parameters	Late presenters (CD4 T cell < 350 and/or AIDS defining event)		*P*
	No (145, 53%)	Yes (130, 47%)	

Age, years*	36 (30–43)	41 (34–50)	0.0001
Gender, male°	120 (83)	105 (81)	0.669
Risk group°			0.020
Homosexual	93 (64)	59 (45)	
Heterosexual	47 (32)	64 (49)	
IDUs	4 (3)	6 (5)	
Other	1 (1)	1 (1)	
Migrants°	30 (21)	43 (33)	0.020
HCV coinfection°	7 (5)	9 (7)	0.382
Calendar year of presentation°			0.385
2007	52 (36)	33 (25)	
2008	32 (22)	29 (22)	
2009	30 (21)	31 (24)	
2010	28 (19)	33 (25)	
January–March 2011	3 (2)		
HIV-RNA log_10_ cp/mL*	4.33 (3.24–4.96)	4.54 (1.77–5.26)	0.884
CD4+ T cells/*μ*L*	538 (441–698)	218 (100–290)	0.0001

**Table:** (b)

T-cells immunophenotypes	Late Presenters (CD4 T cell < 350 and/or AIDS defining event)		*P*
	No (145, 53%)	Yes (130, 47%)	

CD4+ T cells %*	28 (23–35)	14 (10–21)	0.0001
CD8+ T cells %*	45 (38–54)	57 (51–63)	0.0001
CD4+CD127+ cells %*	19 (12–24)	7 (4–12)	0.0001
CD8+CD127+ cells %*	10 (8–15)	11 (7–19)	0.346
CD4+CD95+ cells %*	2 (1–4)	2 (1–3)	0.898
CD8+CD95+ cells %*	2 (1–3)	3 (2–5)	0.0001
CD8+CD38+ cells %*	5 (2–9)	9 (3–18)	0.0001
CD8+CD38+CD45R0+ cells %*	9 (5–16)	15 (10–25)	0.0001

Data are presented as *median, (interquartile range, IQR) and °absolute number, (%). IDUs: injection drug users; migrants: people who were born outside the European community (including people from Eastern Europe, Africa, Asia, and Latin America); HCV: hepatitis C virus.

Comparison between categorical variables was assessed by Pearson's Chi square and between continuous variables by nonparametric Mann-Whitney *U*-test. *P* < 0.05 was considered to denote statistical significance.

**Table:** (a)

Parameters	Advanced HIV disease at presentation		*P*
	(CD4 T cell < 200 and/or AIDS defining event)		
	No (196, 71%)	Yes (79, 29%)	

Age, years*	36 (31–44)	42 (34–52)	0.0001
Gender, male°	158 (81)	67 (85)	0.414
Risk group°			0.044
Homosexual	118 (60)	34 (43)	
Heterosexual	70 (36)	41 (52)	
IDUs	6 (3)	4 (5)	
Other	2 (1)	0	
Migrants°	50 (26)	23 (29)	0.540
HCV coinfection°	10 (5)	6 (11)	0.304
Calendar year of presentation°			0.136
2007	63 (32)	22 (28)	
2008	40 (20)	21 (26)	
2009	50 (25)	11 (14)	
2010	39 (20)	22 (28)	
January–March 2011	4 (3)	3 (4)	
HIV-RNA log_10_ cp/mL*	4.31 (3.17–4.99)	4.68 (1.77–5.37)	0.437
CD4+ T cells/*μ*L*	460 (340–614)	110 (56–192)	0.0001

**Table:** (b)

T-cells immune phenotypes	Advanced HIV disease at presentation		*P*
	(CD4 T cell < 200 and/or AIDS defining event)		
	No (196, 71%)	Yes (79, 29%)	

CD4+ T cells %*	26 (20–32)	10 (7–17)	0.0001
CD8+ T cells %*	48 (41–57)	59 (51–66)	0.0001
CD4+CD127+ cells %*	15 (10–22)	6 (3–11)	0.0001
CD8+CD127+ cells %*	10 (8–15)	12 (8–22)	0.05
CD4+CD95+ cells %*	2 (1–4)	2 (1–3)	0.713
CD8+CD95+ cells %*	2 (1–3)	3 (2–6)	0.0001
CD8+CD38+ cells %*	5 (2–10)	11 (3–25)	0.0001
CD8+CD38+CD45R0+ cells %*	11 (7–19)	14 (10–25)	0.008

Data are presented as *median, (Interquartile Range, IQR) and °absolute number, (%). IDUs: injection drug users; migrants: people who were born outside the European community (including people from Eastern Europe, Africa, Asia, and Latin America); HCV: hepatitis C virus.

Comparison between categorical variables was assessed by Pearson's Chi square and between continuous variables by nonparametric Mann-Whitney* U* test. *P* < 0.05 was considered to denote statistical significance.

**Table 4:** Multivariate logistic regression analysis of the association of demographic and HIV-related characteristics with late presentation.

	Late presenters		*P*
	(CD4 T cell < 350 and/or AIDS-defining event)		
	AOR	95% CI	
(a) Patients' factors			
Age, years	1.052	1.020–1.086	0.001
Risk group°			
Heterosexual	2.435	1.251–4.741	0.009
Other	reference		
Ethnicity			0.017
Migrants	2.543	1.178–5.486	
Non-migrants	reference		

(b) T-cells immune phenotypes			
CD8+ T cells/*μ*L % (each unit more)	1.051	1.022–1.080	0.0001
CD4+CD127+ cells/*μ*L % (each unit more)	0.887	0.857–0.919	0.0001
CD8+CD95+ cells/*μ*L % (each unit more)	0.993	0.874–1.129	0.919
CD8+CD38+ cells/*μ*L % (each unit more)	1.038	0.989–1.089	0.132
CD8+CD38+CD45R0+ cells/*μ*L % (each unit more)	1.003	0.962–1.045	0.893

AOR: adjusted odds ratio; 95% CI, 95% confidence interval. *P* < 0.05 was considered to denote statistical significance.

Other: homosexual, intravenous drug users, and other risks groups.

**Table 5:** Multivariate logistic regression analysis of the association of demographic and HIV-related characteristic with advanced HIV disease at presentation.

	Advanced HIV disease at presentation		*P*
	(CD4 T cell < 200 and/or AIDS-defining event)		
	AOR	95% CI	
(a) Patient factors			
Age, years	1.040	1.010–1.071	0.008
Risk group°			
Heterosexual	1.788	0.901–3.546	0.096
Other	reference		

(b) T-cells immune phenotypes			
CD8+ T cells/*μ*L % (each unit more)	1.051	1.022–1.081	0.001
CD4+CD127+ cells/*μ*L % (each unit more)	0.909	0.878–0.942	0.0001
CD8+CD95+ cells/*μ*L % (each unit more)	1.063	0.841–1.200	0.327
CD8+CD38+ cells/*μ*L % (each unit more)	1.065	1.020–1.112	0.004
CD8+CD38+CD45R0+ cells/*μ*L % (each unit more)	0.955	0.916–0.996	0.032

AOR: adjusted odds ratio; 95% CI, 95% confidence interval. *P* < 0.05 was considered to denote statistical significance.

Other: homosexual, intravenous drug users, and other risks groups.

**Table 6:** Multivariate logistic regression analysis of the association of demographic and HIV-related characteristic with AIDS presentation.

	AIDS presentation (AIDS-defining condition at,		*P*
	or within one month after, HIV diagnosis)		
	AOR	95% CI	
(a) Patient factors			
Age, years	1.015	0.982–1.050	0.374
Gender°			0.001
Male	9.369	2.401–36.551	
Female	reference		
Risk group°			
Heterosexual	4.555	1.937–10.711	0.001
Other	reference		

(b) T-cells immune phenotypes			
CD4+ T cells/*μ*L % (each unit more)	0.996	0.993–0.998	0.002
CD8+ T cells/*μ*L % (each unit more)	0.977	0.931–1.026	0.356
CD4+CD127+ cells/*μ*L % (each unit more)	0.963	0.891–1.042	0.349
CD8+CD95+ cells/*μ*L % (each unit more)	0.994	0.874–1.130	0.922
CD8+CD38+ cells/*μ*L % (each unit more)	1.049	1.004–1.096	0.033
CD8+CD38+CD45R0+ cells/*μ*L % (each unit more)	1.002	0.956–1.052	0.923

AOR: adjusted odds ratio; 95% CI, 95% confidence interval. *P* < 0.05 was considered to denote statistical significance.

Other: homosexual, intravenous drug users, and other risks groups.
