# Supplementary material for: Reduced Central Memory CD4+ T Cells and Increased T-Cell Activation Characterise Treatment-Naive Patients Newly Diagnosed at Late Stage of HIV Infection
Source: AIDS Res Treat. 2011 Oct 27;2012:314849. doi: 10.1155/2012/314849 (PMC3205670; doi:10.1155/2012/314849)
Supplement: Supplementary file 1 — In the Supplementary table 1 we compared demographic parameters and peripheral T lymphocytes immune phenotypes between N-LP and AHD patients (a-c)/AIDS presenters (b-d). The results of the multivariate logistic regression are represented in Supplementary Table 2; we assessed two logistic regression models to explore eventual factors independently assoociated with AHD (model a) and AIDS presentation (model b), using N-LP as the unique comparison group. Significant immune phenotypes in the univariate analysis (CD8+%, CD4+CD127+%, CD8+CD95+%, CD8+CD38+%, CD8+CD38+CD45R0+%) entered the multivariate regression models, adjusted for age and risk group for HIV infection. [file 314849.f1.pdf]

Supplementary table 1

Comparison between AHD/AIDS presenters and N-LP

**Patient's factors**			
**a)**	**N-LP (145, 53%)**	**AHD (79, 29%)**	**p**
Age, years *	36 (30-43)	42 (34-52)	0.0001
Risk group °			0.0001
Heterosexuals	47 (32)	41 (52)	0.016
Other	98 (68)	38 (48)	
Migrants °	30 (21)	23 (29)	0.156
**b)**	**N-LP (145, 53%)**	**AIDS presenters (49, 18%)**	**p**
Age, years *	36 (30-43)	42 (34-52)	0.0001
Risk group °			0.003
Heterosexuals	47 (32)	29 (59)	
Other	98 (68)	20 (41)	
Migrants °	30 (21)	12 (25)	0.577

**T cell immuno-phenotypes**			
**c)**	**NLP (145, 53%)**	**AHD (79, 29%)**	p
CD4+ T cells/µL	538 (441-698)	110 (56-192)	
CD4+ T cells % *	28 (23-35)	10 (7-17)	0.0001
CD8+ T cells % *	45 (38-54)	59 (51-66)	0.0001
CD4+CD127+ cells %*	19 (12-24)	6 (3-11)	0.0001
CD8+CD127+ cells %*	10 (8-15)	12 (8-22)	0.072
CD4+CD95+ cells %*	2 (1-4)	2 (1-3)	0.774
CD8+CD95+ cells %*	2 (1-3)	3 (2-6)	0.0001
CD8+CD38+ cells %*	5 (2-9)	11 (3-25)	0.0001
CD8+CD38+CD45R0+ cells %*	9 (5-16)	14 (10-25)	0.0001
**d)**	**N-LP (145, 53%)**	**AIDS presenters (49, 18%)**	p
CD4+ T cells/µL	538 (441-698)	132 (60-286)	0.0001
CD4+ T cells % *	28 (23-35)	13 (9-19)	0.0001
CD8+ T cells % *	45 (38-54)	59 (50-66)	0.0001
CD4+CD127+ cells %*	19 (12-24)	6 (4-11)	0.0001
CD8+CD127+ cells %*	10 (8-15)	13 (11-25)	0.136
CD4+CD95+ cells %*	2 (1-4)	2 (1-5)	0.408
CD8+CD95+ cells %*	2 (1-3)	3 (2-6)	0.0001
CD8+CD38+ cells %*	5 (2-9)	12 (5-23)	0.0001
CD8+CD38+CD45R0+ cells %*	9 (5-16)	16 (10-25)	0.0001

NOTE (Supplementary table, a-d). Data are presented as * median, (Interquartile Range, IQR) and ° absolute number, (%). Migrants, people who were born outside the European Community (including people from Eastern Europe, Africa, Asia and Latin America).
Comparison between categorical variables were assessed by Pearson's Chi square and between continuous variables by non parametric Mann Whitney U test. $p < 0.05$ was considered to denote statistical significance.

Supplementary table 2

Multivariate logistic regression analysis of the association of demographic and peripheral T lymphocyte immune phenotypes with AHD and AIDS presentation

**Patient factors**	**AOR**	**95% CI**	**p**
**a)**	**Advanced HIV disease at presentation**		
Age, years	1.051	1.012-0.976	**0.010**
Risk group °			
Heterosexual	2.329	0.976-5.56	0.057
Other	reference		
CD8+ T cells/µL % (each unit more)	1.061	1.031-1.093	**0.0001**
CD4+CD127+ cells/µL % (each unit more)	0.837	0.792-0.884	**0.0001**
CD8+CD95+ cells/µL% (each unit more)	1.081	0.934-1.252	0.297
CD8+CD38+ cells/µL % (each unit more)	1.066	1.009-1.126	**0.022**
CD8+CD38+CD45R0+ cells/µL % (each unit more)	0.955	0.993-0.997	**0.0001**
**b)**	**AIDS-presenters**		
Age, years	1.065	1.019-1.113	**0.005**
Risk group °			
Heterosexual	3.176	1.182-8.531	0.022
Other	reference		
CD8+ T cells/µL % (each unit more)	1.025	0.975-1.077	0.335
CD4+CD127+ cells/µL % (each unit more)	0.827	0.759-0.902	**0.0001**
CD8+CD95+ cells/µL% (each unit more)	1.037	0.880-1.221	0.666
CD8+CD38+ cells/µL % (each unit more)	1.078	1.015-1.146	**0.015**
CD8+CD38+CD45R0+ cells/µL % (each unit more)	0.996	0.994-0.999	**0.004**

NOTE (Supplementary table 2, a-b) AOR, adjusted odds ratio; 95% CI, 95% confidence interval. $p < 0.05$ was considered to denote statistical significance.

Other: homosexual, intravenous drug users and other risks groups.
